# Satisfied with teaching? Psychometric properties of the Teaching Satisfaction Scale

**DOI:** 10.4102/ajopa.v5i0.140

**Published:** 2023-12-06

**Authors:** Tyrone B. Pretorius, Anita Padmanabhanunni, Kyle M. Jackson, Brendon D. Faroa

**Affiliations:** 1 Department of Psychology, Faculty of Community and Health Sciences, University of the Western Cape, Cape Town, South Africa

**Keywords:** classical test theory, job satisfaction, psychometric properties, item-response theory, Teaching Satisfaction Scale

## Abstract

Job satisfaction among teachers is a central feature of educational research owing to its benefits for both teachers and students. Compared with their counterparts, teachers satisfied with their roles and responsibilities in the work context demonstrate greater commitment to their organisation, are less likely to leave the profession, and contribute more positively to the educational attainment of their students. Theoretical advances in the study of job satisfaction have emphasised the importance of using stable and robust quantitative measurement tools to facilitate cross-cultural comparisons. This study aims to broaden research on the Teaching Satisfaction Scale (TSS) by examining its psychometric properties through classical test theory (CTT) and Rasch and Mokken analyses. Overall, the three approaches confirmed that the TSS is a unidimensional scale with sound validity and internal consistency. The TSS was also found to be a valuable resource for researchers in different cultural contexts, as it can be used without overburdening teachers and it provides valuable information to support interventions aimed at enhancing job satisfaction.

**Contribution:**

These approaches confirmed that the scale is unidimensional with satisfactory reliability and validity and that the TSS is a valuable resource as it can be used without overburdening teachers and can inform interventions aimed at enhancing job satisfaction.

## Introduction

The COVID-19 pandemic had resulted in the closure of educational institutions globally as a preventive measure. Teachers in most countries had to transition to digital modes of engagement with their students (Jandrić et al., 2022). This process required them to master unfamiliar digital skills and to realign existing pedagogical practices to the digital delivery format (Lizana et al., 2021). The present research was undertaken in the context of the third wave of the disease outbreak in South Africa during 2021. At the time, conventional classroom teaching had resumed, with school teachers reporting increased fear of COVID-19 because of their increased risk of exposure in the school environment (Padmanabhanunni & Pretorius, 2021). Studies also reported increased levels of burnout, anxiety, and depression among this group (Lizana et al., 2021; Padmanabhanunni et al., 2022). In many settings, the absence of organisational support intensified job stressors and contributed to teacher burnout, and fuelled attrition among this population group (Gillani et al., 2022). In sum, teaching is a highly stressful occupation, and teacher well-being has been a public concern both before and during the pandemic (MacDonald & Hill, 2022). Studies on teacher well-being (Bartosiewicz et al., 2022; Sargent & Hannum, 2005) have emphasised the importance of addressing teacher job satisfaction, particularly given its association with improved student learning outcomes, increased job commitment, and reduced risk of leaving the profession. Teaching satisfaction is closely linked to teacher well-being, profession retention, school cohesion, and education quality for students (Toropova et al., 2021). Various scales, such as the Job Descriptive Index (JDI) (Smith, 1969), Minnesota Satisfaction Questionnaire (MSQ) (Brayfield & Rothe, 1951; Weiss et al., 1967), the War’s Job Satisfaction Scale (WJSS) (Warr et al., 1979), and Brayfield–Rothe Job Satisfaction Scale [BRJSS] (Brayfield & Rothe, 1951), have been developed to investigate teacher job satisfaction. However, there have been concerns about the methodological and conceptual shortcomings of these measures (Ho & Au, 2006; Thompson & Phua, 2012). Thompson and Phua (2012) underscore that job satisfaction research is impacted by poor conceptualisation in the development of scales, which has led to a proliferation of instruments that conceptualise job satisfaction affectively but measure it on cognitive domains or that measure only one domain of this construct. The BRJSS, for example, has been criticised (Ho & Au, 2006) for measuring only the affective level of teacher job satisfaction and failing to account for the cognitive dimensions of this construct.

Thompson and Phua (2012) also highlight that there is a lack of clarity regarding the dimensionality of available instruments and an absence of validation studies Scarpello and Hayton (2001), for example, report that although the JDI has been extensively used in job satisfaction research, the dimensionality of the scale has been a source of contention with various researchers reporting five, seven, and nine factor solutions, respectively. Furthermore, certain instruments are overly lengthy which impacts on their utility. For instance, the JDI comprises 72 items and the MSQ comprises 100 items measuring 20 domains of job satisfaction. The length of these surveys makes them both time- and labour-intensive, and prone to respondent bias.

Although it is clear that job satisfaction is a critical component in the realm of teaching, existing tools could lead to skewed or incomplete insights. The length and nature of these scales (e.g. JDI and MSQ) not only strain the participants and researchers, but they also risk capturing superficial or inaccurate representations of true job satisfaction. In some cases, such as with the BRJSS, the scale may overlook fundamental aspects of job satisfaction. The shortcomings of these measures underscore the pressing need for more concise, comprehensive, and validated instruments. To address these gaps, the present study assessed the validity, internal consistency, and responsiveness of the Teaching Satisfaction Scale (TSS). The TSS is a 5-item measure that assesses the extent to which teachers are satisfied with their roles and responsibilities across several domains of their job, such as their work roles, collegial relationships, and interactions with students. The TSS considers both the affective and cognitive dimensions of job satisfaction. When compared to the WJSS and BRJSS, the TSS provides teachers with the tools to form a personal assessment of job satisfaction based on diverse psychological and situational evaluations. It is a simple and convenient global measure of teacher job satisfaction that is based on the concept of deriving pleasure from the overall appraisal of one’s profession in relation to achieving one’s professional values (Ho & Au, 2006). The TSS is grounded in Diener’s Life Satisfaction Scale (Diener et al., 1985), which has been consistently correlated with job satisfaction. It provides a holistic interpretation of different items, that is, a global relevance or impression, which is equivalent to accounting for the subjective experience between one’s real and actual job states, as well as imagined behavioural responses when choosing teaching as a career (Ahammed, 2011). Higher scores on the TSS do not necessarily imply absolute satisfaction and rather indicate the overall impression that teachers may have about their work, thereby accounting for both psychological and situational appraisals across various domains related to job satisfaction (Ho & Au, 2006).

Existing studies have reported that the TSS demonstrates sound internal consistency reliability (i.e. Cronbach’s alpha), construct validity, and criterion-related validity (Ho & Au, 2006). A study on the association of teaching and life satisfaction among teachers in the United Arab Emirates (UAE) found that the TSS demonstrated a satisfactory estimate of internal consistency of 0.70 (Ahammed, 2011). Another study (Parveen & Bano, 2019) exploring the moderating role of Pakistani teachers’ emotions in teaching and the relationship between stress and job satisfaction, reported an alpha coefficient of 0.78 for the TSS. A Chinese study (Ho & Au, 2006) established the validity of the TSS by using WJSS, the BRJSS, the Teaching Stress Inventory, and the Self-Esteem Scale, and reported satisfactory criterion-related and convergent validity with these scales. In a more recent study, Han et al. (2021) examined faculty-related stressors and their relationship with teacher efficacy, engagement, and teaching satisfaction in a large sample of educators from 25 public institutions in East China, and reported satisfactory reliability (α = 0.92). Al Salami et al. (2017), in a study on teachers’ attitudes towards interdisciplinary science, technology, engineering, and mathematics and teaching satisfaction, reported a reliability coefficient of 0.76. Demirtas (2010) investigated the dimensionality of the TSS using Confirmatory Factor Analysis (CFA) and reported a four-factor solution. Yin et al. (2013) in a study on teacher’s emotional intelligence, emotional labour strategies, and teaching satisfaction in China reported a satisfactory internal consistency reliability for the TSS (α = 0.88). In summary, the TSS has demonstrated acceptable reliability coefficients and validity across various teaching contexts globally.

Prior research on the psychometric properties of the TSS (Demirtas, 2010; Han et al., 2021; Ho & Au, 2006; Yin et al., 2013) have largely relied on CFA. While CFA is robust in its capability to evaluate the factorial structure of instruments, it has some limitations. Specifically, CFA primarily focuses on the inter-relationships of items and their underlying latent constructs, but may not delve deeply into individual item characteristics or the specific attributes that influence an individual’s response to an item (Meijer et al., 1990). Additionally, CFA assumes linearity and multivariate normality, conditions that are not always met in real-world data. To address these limitations and provide a comprehensive evaluation of the TSS, the current study aims to extend this work through the use of Item Response Theory (IRT), specifically Rasch and Mokken analysis (i.e. parametric and non-parametric IRT), as well as Classical Test Theory (CTT).

The latter theory posits that all items of a scale contribute equally to an individual’s performance or score on the instrument, whereas IRT distinguishes between people who have varying levels of the underlying traits (Stochl et al., 2012). Models of IRT are generally conceptualised as latent trait models to ‘emphasize that the item response process is explained by constructs hypothesized from the content of the items’ (Franco et al., 2022, p. 2). Item Response Theory enables the comprehensive assessment of items and measures because it considers the pattern of item scores (Franco et al., 2022). For instance, Rasch modelling allows for identifying research participants who respond randomly or idiosyncratically to the items of an instrument (therefore scoring closer to the mean). Mokken analysis is a nonparametric approach which also provides an estimate of internal consistency. This allows it to determine the dimensionality and reliability of an instrument without having to rely on Cronbach’s alpha (Stochl et al., 2012). Mokken analyses is often used as a complementary or secondary analytic approach to examine the extent to which more parametric models such as Rasch models are appropriate and demonstrate adequate performance (Stochl et al., 2012). In sum, these approaches allow for a more nuanced understanding of item functioning, response patterns, and overall test reliability and validity.

The aim of the current study is to investigate the dimensionality and properties of the TSS through IRT, specifically Rasch and Mokken analysis, and CTT. In addition, the Satisfaction with Life Scale (SWLS) (Diener et al., 1985) and the Teaching Identification Scale (TIS) (Brown et al., 1986) are used to assess the criterion-related validity of TSS. It is anticipated that life satisfaction and teaching identification will correlate with teaching satisfaction and demonstrate criterion-related validity when incorporated into the measurement model assessing the factor structure of the TSS.

## Materials and methods

### Participants

We conducted this study during the third wave of COVID-19 from April 2021 to July 2021 when national lockdown was underway in South Africa. Therefore, we were unable to physically interview teachers and had to rely on social media platforms instead. In addition, because South Africa has certain laws that protect personal information, we were unable to use national databases as a sampling frame. Therefore, we used convenience sampling (*n* = 355). Most of the teachers in our sample were women (76.6%) who lived in urban settings (61.7%). The majority were teaching Grades 1 to 7 (61.1%). The teachers in our sample had an average age of 41.9 years (±12.42 years), with an average teaching experience of 15.7 years (±11.74 years).

South Africa had approximately 400 000 teachers in 2021 (Sterne, 2021). Therefore, our sample corresponds to a 5% margin of error (95% confidence level). Despite not being a random sample, our sample was somewhat representative with respect to age, gender, and length of time in the teaching profession. The Organisation for Economic Co-operation and Development (OECD) International Survey of Teaching and Learning conducted in 2019 found that most teachers in South Africa were women (60%), had a mean age of 45 years, and were on average in the teaching profession for 15 years (OECD, 2019). Statistical tests namely one-sample t tests and chi-square demonstrated that our cohort of teachers mirrored these national statistics (teaching experience: *t* = 1.11, *p* > 0.05; age: *t* = 1.68, gender: χ^2^ = 0.06, *p* > 0.05; *p* > 0.05).

### Instruments

Participants completed a brief questionnaire containing demographic items, the TSS (Ho & Au, 2006), the SWLS (Diener et al., 1985), and the TIS (Brown et al., 1986). Both the TIS and the SWLS were included to examine the criterion-related validity of the TSS. Prior research has linked job satisfaction to both professional identity (Scanlan & Hazelton, 2019; Wang et al., 2020) and satisfaction with life (Luque-Reca et al., 2022; Marič et al., 2021).

The TSS comprises five items. The instrument is scored on a 5-point Likert scale that ranges from strongly disagree (1) to strongly agree (5). The scale assesses the extent to which teachers are satisfied with their job. Higher scores on the TSS are indicative of greater job satisfaction. Satisfactory internal consistency estimates for the TSS have been reported in earlier studies, for example, α = 0.78 (Parveen & Bano, 2019) and α = 0.85 (Nalipay et al., 2019).

The TIS assesses the degree to which teachers identify with their profession. The TIS was developed from the Group Identification Scale developed by Brown et al. (1986), but with the word ‘group’ in all items replaced by ‘teacher’. The TIS is a 10-item scale and is scored on a 5-point rating scale that ranges from never (1) to very often (5). Higher scores on the TIS indicate a greater sense of identification with the profession. Satisfactory alpha coefficients have been reported in prior studies using the TIS such as α = 0.82 (Sun et al., 2020) and α = 0.82 (Zeng et al., 2021).

The SWLS measures the cognitive aspect of subjective well-being. It is a five-item scale that is scored on a 7-point Likert-type scale that ranges from strongly disagree (1) to strongly agree (7). Higher scores indicate higher levels of satisfaction with life. The SWLS represents the dominant measure of satisfaction with life and is characterised by high reliability and validity. A South African study reported an alpha coefficient of 0.89 in a sample of students and confirmed the unidimensionality, reliability, and validity of the SWLS in the South African setting (Pretorius & Padmanabhanunni, 2022).

### Procedure

To collect data online, we constructed an online survey through the use of Google Forms. We then sought consent from the administrators of several closed teacher groups on Facebook, a social media platform, to distribute the survey. Representatives from the higher education institution, known as school liaison officers, shared the questionnaire with people they knew within their professional networks.

### Data analysis

To obtain the CTT indices, we used IBM SPSS (Statistical Package for the Social Sciences) for Windows version 28 (IBM Corp., Armonk, NY, USA). To conduct a CFA, we used IBM Amos for Windows (version 27: IBM Corp.). We used the Mokken package (Van Der Ark, 2012) in R (R core team, 2013) for the Mokken analysis. For the Rasch analysis, we used Winsteps 5.1.4 (Linacre, 2023). These three approaches were used to investigate the validity, internal consistency reliability, and the dimensionality of the TSS.

#### Reliability

To examine the reliability of the TSS from the perspective of CTT, we used composite reliability (CR) and Cronbach’s alpha. In Mokken analysis, Mokken scale reliability was used (MSrho). A satisfactory internal consistency reliability typically exceeds 0.70 (Taber, 2017); however it also depends on the purpose of the instrument as high-stakes decisions (such as job selection or student admissions) or instruments used for clinical diagnoses typically require higher reliability.

#### Dimensionality

We examined the factor structure of the TSS with exploratory factor analysis (EFA) and CFA. In the CFA, the following fit indices were used to evaluate model fit: chi-square, the goodness-of-fit index (GFI), the comparative fit index (CFI), the root-mean-square error of approximation (RMSEA), and the Tucker–Lewis Index (TLI). Optimally, the chi-square value should be nonsignificant; although this would be indicative of a perfect fit. Good fit indices would be CFI ≥ 0.90, GFI ≥ 0.95, RMSEA ≤ 0.08, and TLI ≥ 0.90 (Hu & Bentler, 1999).

In Mokken analysis, dimensionality is determined with an automated item selection procedure (AISP), which indicates whether all items load on a single scale. Mokken analysis also yields a scalability coefficient (*H*) that reflects the strength of the scale. In general, an H-coefficient of ≥ 0.50 would indicate a strong scale, whereas an H-coefficient of < 0.40 is indicative of a weak scale (Wind, 2017).

In Rasch analysis, dimensionality is examined using a principal component analysis (PCA) of the unexplained data (standardised residuals) to determine whether there are additional dimensions, other than the latent measure. If there is another dimension (called the first contrast) with an eigenvalue of > 2, the scale is considered to be multidimensional.

#### Validity

Construct validity was examined using item-total correlations (CTT), the item and person index and reliability (Rasch analysis), and the H-coefficient for individual items (H_i_ -Mokken analysis). If the item-total correlations of all items > 0.50 (DeVon et al., 2007) and the H_i_-coefficients > 0.30 (Mokken, 2011), it would indicate that the items contribute to the assessment of the latent variable. In Rasch analysis, the item and person index and reliability indicate whether the items can differentiate between participants with different levels of teaching satisfaction, as well as whether certain items were easier to endorse than others (item-difficulty hierarchy). If the person separation index is > 2 with a person reliability of > 0.80 and the item separation index is > 3 with an item separation reliability of > 0.80, this would confirm that all the items can distinguish between those with different levels of teaching satisfaction and that there is an item-difficulty hierarchy (Linacre, 2023). Rasch analysis also provides infit and outfit mean square (MnSq) statistics which evaluate the degree to which items are aligned to the Rasch model. Mean square values of < 0.50 and > 1.50 reflect misfitting items. Other Rasch indices that were examined include local independence and item hierarchy. Local independence indicates whether there is redundancy among items and whether the responses to items are independent of each other. Local independence was examined with standardised residual item correlations and it is suggested that a standardised residual correlation greater than 0.70 indicates local dependency (Linacre, 2020). Item hierarchy refers to the ordering of items in terms of the likelihood of being endorsed. The item ordering is expressed as logits (log odds units) with higher values indicating that the item is more difficult to endorse. Item ordering was also visually inspected with a Wright map (person and item map) where persons’ level of the latent variable (teaching satisfaction) is plotted on the left and item ordering on the right side of the plot. Mokken analysis provides an indication (called a Crit value) of whether an item can distinguish between high and low scorers (monotonicity), and whether an item that is harder to endorse for one person is harder to endorse for all participants, referred to as invariant item ordering (IIO). A Crit value of > 80 provides an indication of a critical violation of monotonicity and IIO.

Convergent validity is confirmed if the average variance extracted (AVE) is greater than 0.50, if the factor loadings are significant, and if AVE < CR (Ghadi et al., 2012; Hajjar, 2018; Posch et al., 2019). With regards to discriminant validity, teaching satisfaction should account for more of the variance in the individual items (AVE) compared with the variance that it shares with related constructs, which is assessed with average shared variance (ASV) and maximum shared variance (MSC). With regards to criterion-related validity, both life satisfaction and teaching identification, which are presumed to be related to teaching satisfaction, were included in the measurement model that was used to assess the factor structure of the TSS (see Figure 2).

### Ethical considerations

This study was undertaken in line with the Declaration of Helsinki. Ethical approval for this study was obtained from the Humanities and Social Sciences Ethics Committee of the University of the Western Cape (Reference number: HS21/3/8). Participation was voluntary, and all participants were assured of their conventionality and provided informed consent before they were allowed to proceed with the electronic survey.

## Results

Table 1 lists the Mokken, Rasch, and CTT indices at the item level as well as the inter-item correlations. The range of the inter-item correlations was between 0.49 and 0.80 and did not exceed 0.85, which would indicate item redundancy (Paulsen & BrckaLorenz, 2017).

**TABLE 1:** Inter-item correlations and classical test theory, Rasch, and Mokken indices at the item level.

Items and indices	Items				
	1	2	3	4	5
1. Being a teacher is close to ideal	-	-	-	-	-
2. Excellent conditions for teacher	0.49**	-	-	-	-
3. Satisfied with being a teacher	0.80**	0.54**	-	-	-
4. Have the most important things	0.60**	0.59**	0.65**	-	-
5. Would change almost nothing	0.56**	0.50**	0.57**	0.56**	-
Mean	3.86	3.08	3.73	3.39	3.19
SD	1.07	1.18	1.07	1.10	1.30
Item-total correlations	0.74	0.63	0.78	0.73	0.65
Factor loadings	0.85	0.75	0.88	0.83	0.77
Infit MnSq	0.91	1.17	0.74	0.86	1.27
Outfit MnSq	0.84	1.27	0.70	0.87	1.34
Item hierarchy	−0.99	0.84	−0.64	0.18	0.61
H_i_	0.67	0.58	0.69	0.66	0.59
*Crit* values for monotonicity	0	0	0	0	0
*Crit* values for IIO	0	29	0	0	31

SD, standard deviation; MnSq, mean square; H_i_, scalability coefficient for individual items; IIO, invariant item ordering.

** , *p* < 0.001.

**FIGURE 1:**
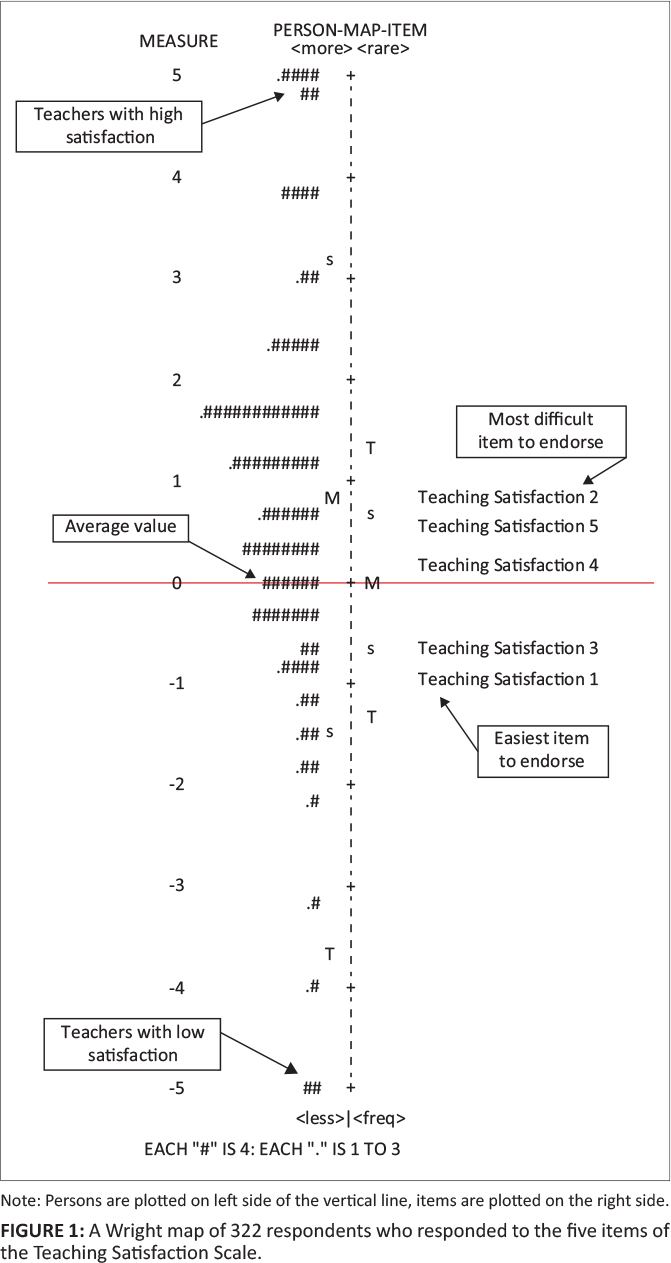
A Wright map of 322 respondents who responded to the five items of the Teaching Satisfaction Scale.

The correlations between the items and the total score were all significant and > 0.50. A single factor was extracted in EFA which explained 60% of the variance. The factor loadings ranged between 0.75 and 0.88, and all were statistically significant. In terms of mean square, no misfitting items were observed, as infit MnSq values ranged between 0.74 and 1.27 and outfit MnSq values ranged between 0.70 and 1.34. Table 1 also indicates that item 2 was the most difficult to endorse while item 1 was the easiest to endorse. This was confirmed by the ordering of items on the right side of the Wright map in Figure 1. The Wright map also reflects that most participants were above the average (0) level of satisfaction with some extreme outliers at both high and low level of teaching satisfaction. With regard to local item dependency, the analysis of standardised residual correlations indicated that there were dependencies only between items 1 and 3 (0.28); however as this was way below the recommended 0.70, it can be safely assumed that no item displayed problematic local independence. All H-coefficients for individual items (*Hi*) were above the threshold of 0.30 and ranged between 0.58 and 0.69. All Crit values for monotonicity were zero, indicating no violations of monotonicity. Although a single Crit value of 29 was observed for item 2, it was well below the threshold of 80.

**FIGURE 2:**
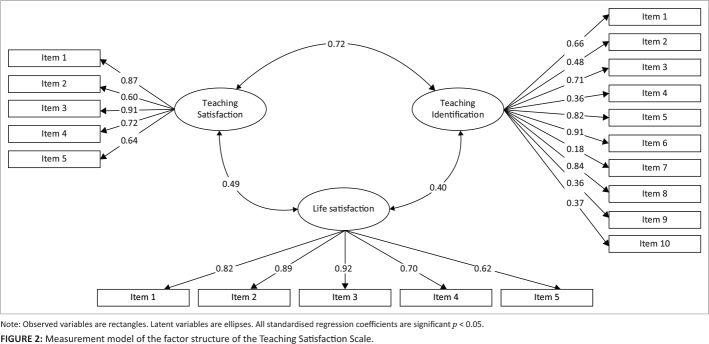
Measurement model of the factor structure of the Teaching Satisfaction Scale.

Figure 2 depicts the measurement model that was used to evaluate the structure of the TSS and its relationship with teaching identification and life satisfaction through structural equation modelling (SEM). In this model, the items of various scales are presented as observed variables, and teaching satisfaction, teaching identification, and life satisfaction are presented as latent variables. In general, the GFIs for this model may be deemed satisfactory, χ^2^ = 302.59, *p* > 0.05, GFI = 0.92, CFI = 0.96, TLI = 0.95, RMSEA = 0.05. As indicated by the model in Figure 2, there was a significant association between teaching satisfaction, on the one hand, and life satisfaction (*r* = 0.49, *p* = 0.012) as well as teaching identification (*r* = 0.72, *p* = 0.003), on the other hand.

Table 2 lists the Mokken, Rasch, and CTT indices at the scale level. Automated item selection procedure in Mokken analysis reflected that all items loaded on a single dimension. Table 2 further shows that all reliability indices were at a satisfactory level: α = 0.87, CR = 0.91, and MSrho = 0.88. Average variance extracted was above 0.50 and greater than MSV and ASV. The person and item index and reliability exceeded the recommended thresholds, and PCA did not identify any additional dimensions with an eigenvalue > 2. The eigenvalue of the Rasch dimension was 8.26 and the amount of variance explained was 62.3% and those for the first contrast was 1.78% and 13.4%, respectively. The H-index in Mokken analysis indicated a very strong scale.

**TABLE 2:** Classical test theory, Rasch, and Mokken indices for the Teaching Satisfaction Scale at the scale level.

Index	Value	Suggested cut-off
Cronbach’s alpha	0.87	> 0.70
Composite reliability	0.91	> 0.70
Average variance extracted	0.67	> 0.50
Maximum shared variance	0.33	< AVE
Average shared variance	0.27	< AVE
Item separation reliability (Rasch)	0.99	> 0.80
Item separation index (Rasch)	8.27	> 3
Person separation reliability (Rasch)	0.82	> 0.80
Person separation index (Rasch)	2.17	> 2
Eigenvalue of likely second dimension (Rasch)	1.78	< 2
Scale H (Mokken)	0.64	> 0.50
Mokken scale reliability (MS_rho_)	0.88	> 0.70

AVE, average varience extracted.

## Discussion

Teacher job satisfaction is a central component of educational research owing to its potential benefits for both teachers and students (Bartosiewicz et al., 2022). According to multiple studies (Gillani et al., 2022; Jandrić et al., 2022), teachers who are satisfied with their jobs demonstrate greater organisational commitment and are less inclined to leave their profession. Teaching satisfaction correlates with the gratification of higher-order needs, particularly in positive social relationships with students, parents, and co-workers (Pretorius et al., 2022). Teachers who experience a sense of dissatisfaction with their jobs may exhibit behaviours that counter the goals of the educational system, such as frequent absenteeism and the lack of commitment to students’ learning needs. In South Africa, teacher job dissatisfaction has been correlated to high workloads, limited opportunities for growth, and job insecurity (Nomatolo, 2022). Teacher dissatisfaction has also been linked to common mental health disorders such as depression, anxiety, and burnout as well as physical health conditions including hypertension and fatigue (Padmanabhanunni & Pretorius, 2022; Rothmann & Fouché, 2018). Therefore, given the central role of teaching satisfaction in promoting and maintaining the well-being of teachers and the academic attainment of students, measuring the extent to which teachers are satisfied with their job has been identified as a critical area of study. Theoretical advances in the study of job satisfaction (Judge et al., 2020) have under-scored the need to use stable and robust quantitative measurement tools to enable cross-cultural comparisons.

The current study employed CTT and item-response theory to assess the psychometric properties of the TSS. Overall, our three approaches confirmed that the TSS is a unidimensional scale with satisfactory reliability and validity. Exploratory factor analysis, CFA, PCA in Rasch analysis and AISP in Mokken analysis also confirmed that the TSS is essentially unidimensional. Exploratory factor analysis extracted a single factor that accounted for a sufficient amount of variance while the CFA established that a one-factor model was a sound fit for the data. In addition, the H-coefficient demonstrated that the scale can be regarded as strong. In terms of dimensionality, these results confirmed those of the original authors of the scale (Ho & Au, 2006), indicating that the scale is unidimensional, a conclusion based on EFA. They also confirmed the results of the CFA conducted by Nalipay et al. (2019).

Similar to previous studies that reported sound internal reliability coefficients (Ho & Au, 2006; Nalipay et al., 2019; Parveen & Bano, 2019), the TSS also demonstrated satisfactory Cronbach’s alpha, Mokken scale reliability, and CR. It also demonstrated adequate construct validity, convergent validity, discriminant validity, and criterion-related validity. In particular, the item-total correlations and H-coefficients of scale items (Mokken analysis) established the construct validity of the TSS by confirming that all scale items contributed to the measurement of the underlying variable. The results of the Rasch and Mokken analysis further demonstrated that the TSS items differentiated between low levels and high levels of teaching satisfaction through person index and reliability (Rasch) and monotonicity (Mokken). Moreover, the item index and reliability in Rasch analysis confirmed the existence of a hierarchy of item-difficulty. In addition, IIO in Mokken analysis indicated the absence of scale items that teachers with similar levels of teaching satisfaction might endorse differently. Lastly, the infit and outfit MnSq values in Rasch analysis confirmed the absence of misfitting items.

Average variance extracted was greater than 0.50 and CR, all factor loadings were significant, thus confirming convergent validity. The results also indicated that teaching satisfaction accounted for more of the variance in the individual items (AVE) as opposed to the variance that it shared with other related variables (i.e. MSV and ASV), namely teaching identification and life satisfaction, thus confirming the notion of discriminant validity. Lastly, criterion-related validity was confirmed by the significant association between teaching satisfaction on the one hand and life satisfaction and teaching identification on the other hand. These validity results further supported the validity data provided by Ho and Au (2006).

The results of the study have potential implications. The finding regarding the unidimensionality of the TSS is consistent with prior research and reinforces the potential of the instrument to be a universal measure of job satisfaction among teachers. The results of the Rasch and Mokken analysis suggest that the TSS can discern between varying degrees of teaching satisfaction. This means that institutions can potentially use the TSS to identify specific needs or concerns among educators with varying levels of teaching satisfaction. As the TSS is a valid and reliable tool, it can also be used to make informed decisions about teacher-wellbeing, intervention strategies, and policy decisions aimed at enhancing the overall teaching and learning environment. Furthermore, given the associations between satisfaction with life, teacher identification, and teaching satisfaction, it may be worthwhile for policy makers to consider holistic well-being programmes that enhance overall life satisfaction, potentially improving teacher retention rates.

### Limitations of the study

This study has certain limitations. Firstly, most of the teachers in our sample were women from a single province. Therefore, our results may not be generalisable to other populations. The teaching profession is disproportionately female. Nevertheless, gender can play a significant role in shaping one’s experiences, perceptions, and challenges in the teaching profession. Men might therefore have different experiences, stressors, or satisfaction levels based on the interplay of societal norms, educational policies, and individual factors. Therefore, the predominantly female sample may not capture the full spectrum of teaching experiences. Future studies should therefore include a larger and more diverse sample. This can also include teachers with varying levels of experience (e.g. mid-career versus novice teachers) and those with a spectrum of teaching experiences based on the school setting (e.g. disadvantaged versus more privileged schools). Secondly, we used a self-report instrument, which may have introduced response bias and social desirability bias. These types of biases have the potential to artificially inflate the instruments reliability or validity metrics, and the underlying factor structure of the instrument may not be accurately depicted (Weigold et al., 2018). Thirdly, it is possible that only teachers who were interested in the study and had access to information and communication technologies (ICTs) responded to the survey. These teachers may already be positively inclined toward the topic or have specific experiences that motivated them to participate. This means that the results may not represent the broader population of teachers but rather a subset with particular views or experiences. Furthermore, by potentially excluding teachers who may not have access to ICT, the survey may not capture the perspectives of those who may face the most significant challenges in integrating technology into education or have different experiences and needs. Therefore, future research incorporating complementary approaches is recommended to confirm our findings.

## Conclusion

According to both CTT and IRT, the TSS is a unidimensional scale with satisfactory reliability and validity. Our findings suggest that the TSS can be used across different settings (e.g. for routine screening purposes) and cultural contexts. The TSS can also be used as a valuable resource for researchers, as it can be used without overburdening teachers and can potentially provide valuable information to inform interventions aimed at enhancing job satisfaction.
